# A Giant Acquired Dacryocystocele in Congenital Nasolacrimal Duct Obstruction

**DOI:** 10.22336/rjo.2025.92

**Published:** 2025

**Authors:** Prabrisha Banerjee, Abhishek Gupta, Prabhakar Singh, Abhishek Verma, Shreeyaa Mohanty, Mahuya Chattopadhyay

**Affiliations:** 1 All India Institute of Medical Sciences, Kalyani, West Bengal, India

**Keywords:** acquired dacryocystocele, Rosenmuller valve, medial canthal mass, nasolacrimal duct cyst, congenital nasolacrimal duct obstruction, CNLDO = Congenital Nasolacrimal Duct Obstruction, ROPLAS = Regurgitation on pressure over lacrimal sac, CT = Computed Tomography, NLD = Nasolacrimal duct, CDC = Congenital Dacryocystocele, DCR = Dacryocystorhinostomy

## Abstract

**Introduction:**

Dacryocystoceles are cystic dilatations of the lacrimal sac and nasolacrimal duct (NLD) due to the outflow obstruction at the Rosenmuller valve and the Hasner valve. Dacryocystoceles are rare in adults.

**Materials and methods:**

A retrospective case report.

**Case presentation:**

We describe the first case of an acquired dacryocystocele in a patient with bilateral congenital nasolacrimal duct obstruction (CNLDO). A 25-year-old treatment-naïve bilateral CNLDO male presented with a superolateral dystopia of the left globe, proptosis, and a non-tender cystic mass at the left medial canthal region for the past six months. Computed tomography (CT scan) revealed a cystic enlargement of the left lacrimal sac with a distended NLD forming a nasolacrimal duct mucocele. Bilaterally, external DCR was performed. Histopathology was consistent with chronic dacryocystitis.

**Discussion:**

Dacryocystoceles are rare in adults. They are either idiopathic or secondary to dacryocystitis, trauma, or dacryocystorhinostomy (DCR) surgery. In this case, the long-standing NLD obstruction has caused distention of the NLD and the lacrimal sac, leading to chronic fibroinflammatory changes in the Rosenmuller valve.

**Conclusion:**

Chronic fibroinflammatory changes in adult patients with untreated congenital nasolacrimal duct obstruction can cause dacryocystocele formation.

## Introduction

Dacryocystoceles are cystic dilatations of the lacrimal sac and NLD due to the outflow obstruction at the Rosenmuller valve proximally and the Hasner valve distally. It is found in 0.1%- 1.3% of patients with congenital nasolacrimal duct obstruction (CNLDO) and shows a female preponderance [**1,2**]. Dacryocystoceles are rare in adults. They are idiopathic or secondary to dacryocystitis, trauma, or dacryocystorhinostomy (DCR) surgery [**3**]. We describe the first case of an acquired dacryocystocele in a patient with bilateral CNLDO. The patient’s informed consent was obtained, and the case report adhered to the tenets of the Declaration of Helsinki.

## Materials and methods

A retrospective case report.

## Case presentation

**Fig. 1:**
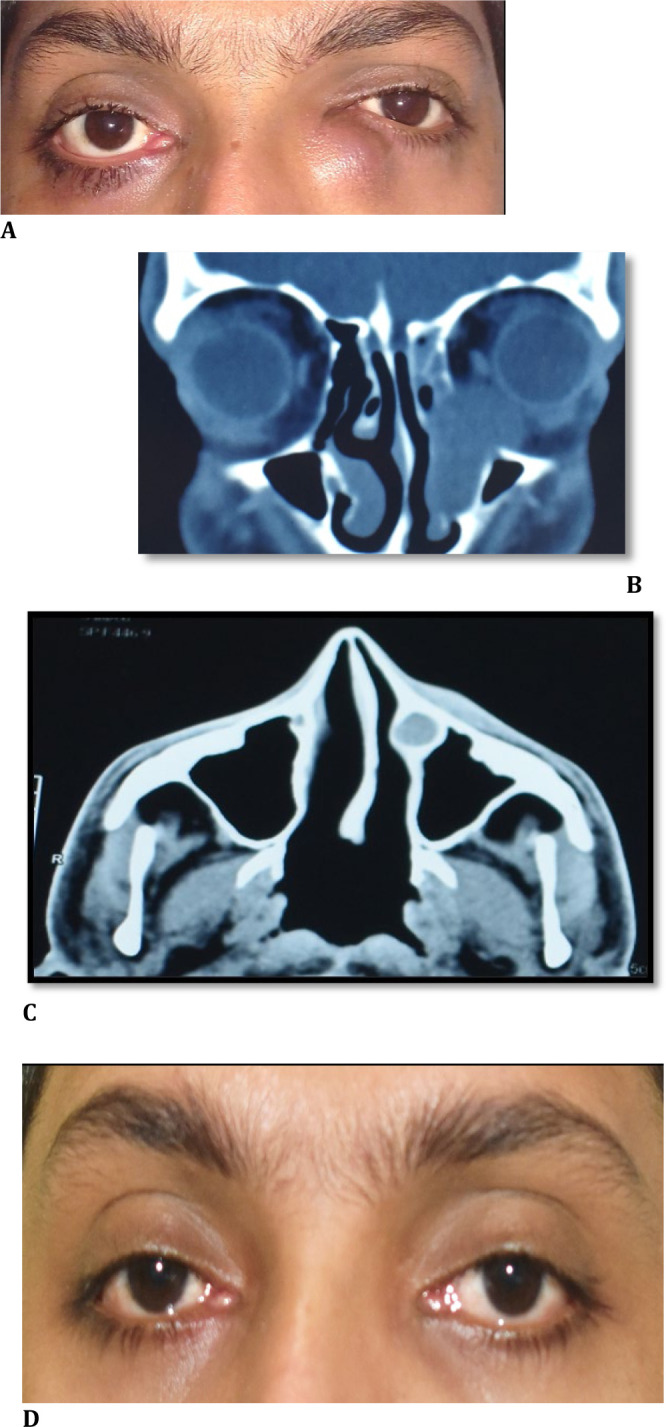
**A**. Clinical picture showing medial canthal swelling and superolateral dystopia in the left eye; **B, C**. CT scan displaying the expansion of the cystic lacrimal sac into the orbit with a dilated nasolacrimal duct; **D**. Patient at one-year follow-up visit

A 25-year-old male presented with a displaced left globe and a swelling at the left medial canthal region for the past six months. He had watering in both eyes since birth. He was diagnosed with bilateral CNLDO at six months of age, and Crigler massage was advised. There was no associated respiratory distress. When symptoms did not resolve, probing was planned, but the parents were unwilling to undergo surgical intervention. There was no history of acute dacryocystitis. On examination, a well-defined, large, non-tender cystic mass at the left medial canthus was noted, leading to superolateral dystopia and 2mm proptosis (**Fig. 1A**). Both eyes’ visual acuity, ocular motility, anterior segment evaluation, and fundoscopy were normal. The regurgitation (ROPLAS) test was positive on the right and negative on the left. On the left side, syringing was not patent, and probing revealed a common canaliculus block. Computed tomography (CT scan) displayed a hypodense cystic enlargement of the lacrimal sac continuing as the distended NLD and forming a nasolacrimal duct mucocele (**Fig. 1B, C**). Nasal endoscopy revealed a deviated nasal septum to the left with a bony spur impinging on the inferior turbinate. A bilateral external DCR was performed. Trypan blue was injected into the left lacrimal sac to delineate the entire sac. A yellow viscous fluid escaped on piercing the sac, which was sent for histopathological and microbiological evaluation. After fashioning the sac flap, the remaining cystic lacrimal sac tissue was carefully removed and sent for histopathology. Mitomycin C (0.02%) was applied, and a Silastic intubation was performed. Histopathological findings were consistent with chronic dacryocystitis. No microbial growth was observed in the specimen. Epiphora resolved, and the patient was doing well at the 1-year follow-up visit (**Fig. 1D**).

## Discussion

Dacryocystoceles result from obstruction of the Hasner valve, which distends the nasolacrimal duct (NLD) and the lacrimal sac, leading to compression and functional failure of the Rosenmuller valve [**1,2**]. The patient had bilateral CNLDO. However, the cystic mass appeared at the left medial canthus 24 years later. Chronic inflammation of the Rosenmuller valve secondary to long-standing distal obstruction is the likely cause [**4**]. The chronically distended NLD led to bone remodeling in that area. The expansion of the sac into the orbital cavity caused proptosis and dystopia. Usually, a congenital dacryocystocele (CDC) manifests as a fluctuant, compressible, bluish swelling containing clear to yellowish fluid. While acquired dacryocystoceles are firmer, the bluish hue from the Tyndall phenomenon is attenuated by the thicker tissue planes and contains viscous liquid derived from the sac’s mucosal epithelium [**5**]. The differential diagnosis here can be lacrimal sac mucocele, lacrimal sac diverticulum, ethmoidal mucocele, dermoid cyst, epidermoid cyst, and lacrimal sac neoplasm [**2,5**]. Radioimaging helps in the definitive diagnosis. Nasal endoscopy aids in surgical planning. In this case, the external DCR provided adequate access to remove the enlarged sac tissue, as confirmed by trypan blue injection. Bicanalicular intubation was done due to the inflammatory changes in the canaliculi [**6**].

## Conclusion

To conclude, chronic fibroinflammatory changes in patients with persistent congenital nasolacrimal duct obstruction can lead to dacryocystocele formation. Imaging and nasal endoscopy facilitate proper management. DCR surgery is the treatment of choice. Silastic intubation increases its success rate. Timely and appropriate management of congenital nasolacrimal duct obstruction is recommended to avoid secondary dacryocystocele formation.
